# Alleviation of Lead Toxicity by 5-Aminolevulinic Acid Is Related to Elevated Growth, Photosynthesis, and Suppressed Ultrastructural Damages in Oilseed Rape

**DOI:** 10.1155/2014/530642

**Published:** 2014-02-11

**Authors:** Tian Tian, Basharat Ali, Yebo Qin, Zaffar Malik, Rafaqat A. Gill, Shafaqat Ali, Weijun Zhou

**Affiliations:** ^1^Institute of Crop Science and Zhejiang Key Laboratory of Crop Germplasm, Zhejiang University, Hangzhou 310058, China; ^2^Crop Production Bureau, Zhejiang Provincial Department of Agriculture, Hangzhou 310020, China; ^3^College of Environmental and Resource Sciences, Zhejiang University, Hangzhou 310058, China; ^4^Department of Environmental Sciences, Government College University, Faisalabad 38000, Pakistan

## Abstract

Lead (Pb) is a widely spread pollutant and leads to diverse morphological and structural changes in the plants. In this study, alleviating role of 5-aminolevulinic acid (ALA) in oilseed rape (*Brassica napus* L.) was investigated with or without foliar application of ALA (25 mg L^−1^) in hydroponic environment under different Pb levels (0, 100, and 400 µM). Outcomes stated that plant morphology and photosynthetic attributes were reduced under the application of Pb alone. However, ALA application significantly increased the plant growth and photosynthetic parameters under Pb toxicity. Moreover, ALA also lowered the Pb concentration in shoots and roots under Pb toxicity. The microscopic studies depicted that exogenously applied ALA ameliorated the Pb stress and significantly improved the cell ultrastructures. After application of ALA under Pb stress, mesophyll cell had well-developed nucleus and chloroplast having a number of starch granules. Moreover, micrographs illustrated that root tip cell contained well-developed nucleus, a number of mitochondria, and golgi bodies. These results proposed that under 15-day Pb-induced stress, ALA improved the plant growth, chlorophyll content, photosynthetic parameters, and ultrastructural modifications in leaf mesophyll and root tip cells of the *B. napus* plants.

## 1. Introduction

Environmental pollutants like heavy metals are toxic even at low concentration. Lead (Pb) is amongst one of the most toxic pollutants which is not an essential nutrient for plants [1]. The contamination of Pb in environment is of major ecological alarm due to its long persistent nature that causes risk effects on human health through the food chain [2]. Lead accumulation is recognized to cause highly deleterious effects on growth and yield of plants [3]. It was reported that Pb significantly suppressed the root elongation in mesquite (*Prosopis* sp.) [4]. Plant growth retardation under Pb stress may be due to nutrient imbalance, disturbed photosynthesis, obstacle in electron transport system, and inadequate levels of carbon dioxide due to stomatal closure [5, 6]. It is documented that Pb prohibits the nutrient uptake, net photosynthetic rate, and cellular respiration and causes damage to cell membrane [7]. It has been stated that photosynthesis inhibition is a well-recognized symptom of Pb toxicity [8]. Lately, Jiang and Liu [9] proposed that exposure of Pb for 72 hr to *Allium sativum *roots induced ultrastructural changes, that is, loss of cristae, mitochondrial swelling, dictyosomes, endoplasmic reticulum vacuolization, impairment into lamellar organization of the chloroplast, and cell division [10]. Thus, it is very important to exploit the potential of oilseed rape plant against Pb stress.

The plant growth regulators (PGRs) are mostly used to alleviate abiotic stress in plants. The 5-aminolevulinic acid (ALA), one of the most vital plant growth regulators, is an essential precursor for the formation of tetrapyrroles like protochlorophyllide (which is converted into chlorophyll when exposed to light) [11]. In our previous research, ALA had been found to promote growth of *B. napus* under cadmium (Cd) stress [12, 13]. Similarly, Zhang et al. [14] applied the ALA in oilseed rape plants and suggested that it alleviates the toxicity of herbicides. Wang et al. [15] delineated that treatment of *B. campestris* L. with ALA yielded a positive effect on the seedlings growth. Furthermore, ALA improved the plant growth in terms of biomass of different plant parts under salinity-stress conditions [16] and ALA is the major photosynthetic light harvesting pigment [17]. Recently, it has been documented that root morphology was exalted under the combined treatment of ALA and Cd in *B. napus *[18].

It is evident from a number of previous reports that ALA, like other known PGRs, is effective in counteracting the injurious effects of various abiotic stresses on plants, but the mechanisms how it regulates growth under stressful cues are not fully elucidated yet. The present research was undertaken to investigate the changes in the level of plant growth, photosynthetic activity, and Pb uptake due to ALA application on *B. napus* under Pb stress. It is hypothesized that ALA regulates the growth, photosynthetic attributes, chlorophyll contents, and ultrastructural changes under Pb stress conditions in *B. napus* L.

## 2. Materials and Methods

### 2.1. Plant Material and Treatments

For the experiment, *B. napus* L. cv. ZS 758 seeds were obtained from Zhejiang University. The seeds were grown in plastic pots (170 mm × 220 mm) which were filled with peat moss. At five-leaf stage, uniform seedlings were plugged into plate holes on plastic pots (five plants per pot) containing Hoagland nutrient solution [19], in which concentration of KH_2_PO_4_ was kept as minimum as 0.01 mM in order to avoid precipitation of lead (Pb). The composition of Hoagland nutrient solution was as follows (in *μ*mol L^−1^): 3000 KNO_3_, 2000 Ca(NO_3_)_2_·4H_2_O, 1000 MgSO_4_·7H_2_O, 10 KH_2_PO_4_, 12 FeC_6_H_6_O_7_, 500 H_3_BO_3_, 800 ZnSO_4_·7H_2_O, 50 MnCl_2_, 300 CuSO_4_·5H_2_O, and 100 Na_2_MoO_4_. The pH was maintained at 5.5 with 1 M solution of NaOH or HCl. Aeration was given continuously through air pump in the nutrient medium. The nutrient solution was changed after every four days.

After two weeks, solutions were supplied to required Pb as Pb(NO_3_)_2_ concentration (0, 100, and 400 *μ*M) and simultaneously plants were sprayed with an aqueous solution of ALA (Cosmo Oil Co. Ltd., Japan) at concentrations of 0 and 25 mg L^−1^ ALA. The spray was done on the lower as well as the upper leaf surfaces with a hand-held atomizer [20]. ALA was applied as a foliar spray at a rate of 20 mL of formulated solution/plant. After the five days of first spray, subsequent application was followed. The control plants were sprayed with distilled water. Fifteen days after treatment, all morphological data and photosynthetic gas exchange parameters were recorded. Samples for chlorophyll contents and microscopic studies of leaf mesophyll and root tip cell were collected as described next.

### 2.2. Plant Morphological Parameters

After 15 days of treatment, harvested plants were separated into leaf, stem, and root. The plant height, stem length, root length, and leaf area were recorded simultaneously. Root surface area, volume, diameter, and number of root tips of randomly selected plants were determined using root automatism scan apparatus (MIN MAC, STD1600^+^), equipped with WinRHIZO software offered by Regent Instruments Inc.

### 2.3. Photosynthetic Attributes

Photosynthetic parameters were analyzed 15 days after treatment by LiCor-6400 portable photosynthesis system (Li-Cor Inc., Lincoln, NE, USA). All the photosynthetic attributes were measured on the intact topmost fully expanded leaf after 2 hr of acclimatization in a growth cabinet, at a temperature of 18°C under a light intensity of 1,000 *μ*mol m^−2^ s^−1^, relative humidity of 60%, and took at least eight readings per treatment [21]. Chlorophyll contents in *B. napus* leaves were estimated according to Porra et al. [22] and Pei et al. [23].

### 2.4. Analysis of Pb Concentration

Pb contents in different plant parts were determined with the method of Hsu and Kao [24]. The tissue samples were dried at 65°C for 24 hr and then ashed in Muffle furnace at 550°C for 20 hr. Then the ash incubated was with 31% HNO_3_ and 17.5% H_2_O_2_ at 70°C for about 2 hr, and then this was dissolved in distilled water. Pb concentration was determined by using spectrophotometer (AA-6800, Shimadzu Co. Ltd., Japan).

### 2.5. Microscopic Study

After 15 days of treatment, topmost leaf fragments without veins and root tips were collected from randomly selected plants and then fixed overnight in 4% glutaraldehyde (v/v) in 0.1 M PBS (sodium phosphate buffer, pH 7.4) followed by three times washing with the same PBS. Samples were postfixed in 1% OsO_4_ (osmium (VIII) oxide) for 1 hr, washed three times in 0.1 M PBS (pH 7.4) with 10 min interval between each washing. Then with 15–20 min interval, the samples were dehydrated in a graded series of ethanol (50%, 60%, 70%, 80%, 90%, 95%, and 100%) and at the end washed by absolute acetone for 20 min. The samples were then infiltrated and embedded in Spurr's resin overnight. After heating at 70°C for 9 hr, ultrathin sections (80 nm) of specimens were prepared and mounted on copper grids for viewing by a transmission electron microscope (JEOL TEM-1230EX) at an accelerating voltage of 60.0 kV.

### 2.6. Statistical Analysis

The SPSS version 16.0 (SPSS, Chicago, IL, USA) was used to analyze the data. A two-way variance analysis (ANOVA) was carried out, followed by the Duncan's multiple range test.

## 3. Results

### 3.1. ALA Ameliorated Pb-Induced Inhibition of Plant Growth

The data regarding plant height, stem length, root length, and number of leaves per plant under different treatments of ALA and Pb is given in Table 1. The results showed that Pb toxicity significantly inhibited the plant growth as compared to the control. The Pb toxicity at higher level (400 *μ*M) visibly reduced the plant length by 40.24%, shoot length by 56.08%, root length by 36.61% and number of leaves per plant by 46.49% as compared to control plants. Foliar applied ALA at 25 mg L^−1^ significantly improved the plant growth parameters under Pb toxicity. Moreover, application of ALA alone did not show any change in the plant growth; rather, it alleviated the adverse effects of Pb under Pb stress conditions (Table 1). The effects of ALA and Pb at different treatments on root morphology of *B. napus* have been shown in Table 2. The data clearly demonstrated that root diameter, root surface area, root volume, and number of root tips significantly were reduced as Pb concentrations increased in the solution culture. Higher concentration of Pb (400 *μ*M) alone significantly reduced the root diameter by 47.56%, root surface area by 92.63%, root volume by 40.23%, and number of root tips by 32.42% in contrast to control plants. However, ALA increased the root morphology and this increase was in root diameter by 24.93% and 83.72%, in root surface area by 16.75% and 15.70%, in root volume by 30.91% and 40.15% and in number of root tips by 15.08% and 27.97% under different concentrations of Pb (100 and 400 *μ*M, resp.) as compared to their respective controls. Moreover, exogenous ALA alone significantly enhanced the root morphology except root volume as compared to control.

### 3.2. Photosynthetic Parameters

**Figure 1:**
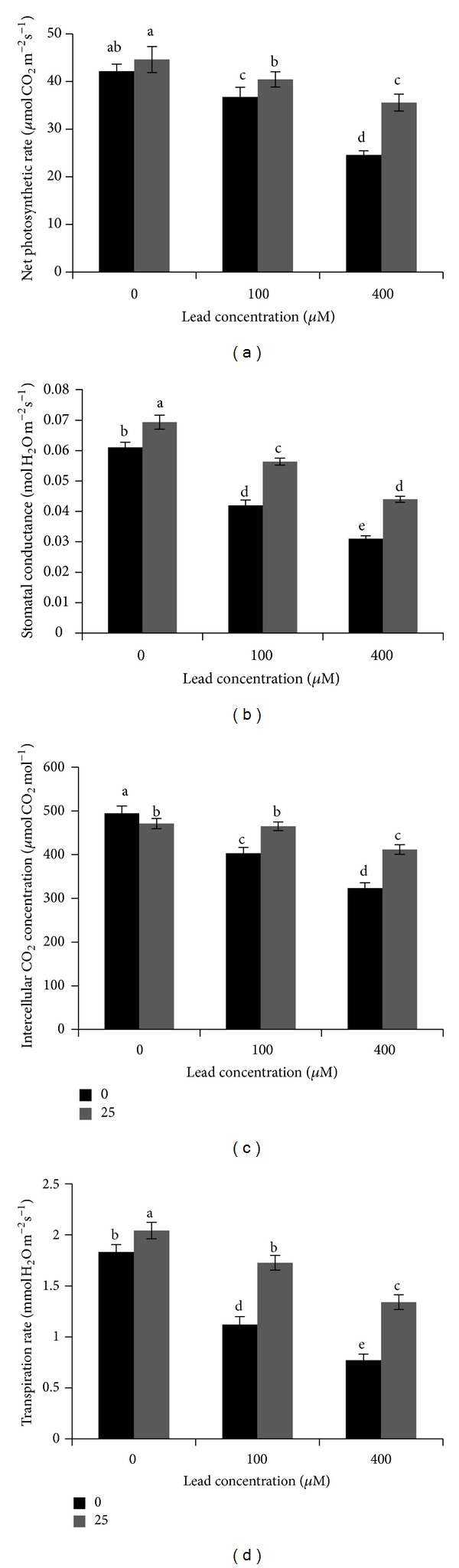
Effects of treatments of 5-aminolevulinic acid (ALA) (0, 25 mg L^−1^) and lead (Pb) (0, 100, and 400 *μ*M) on (a) net photosynthetic rate, (b) stomatal conductance, (c) intercellular CO_2_ concentration, and (d) transpiration rate of the youngest fully expanded leaf in *Brassica napus* cv. ZS 758. Values are means of eight readings ± SD. Columns marked with the same lowercase letters are not significantly different by the LSD test at *P* ≤ 0.05.

The data regarding chlorophyll attributes under different treatments of ALA and Pb are expressed in Table 3. The results showed that Pb stress alone visibly reduced the Chl a, Chl b, total Chl, and carotenoid contents in the leaves of *B. napus* as compared to control. Foliar applied ALA did not show any impact on Chl contents under Pb stress conditions; however, it significantly increased the total Chl contents by 11.88% under higher concentration of Pb (400 *μ*M), as compared to its respective control. Moreover, the higher chlorophyll values were found under ALA alone conditions. The effects of different treatments of ALA and Pb on photosynthetic parameters have been presented in Figure 1. The data depicted that Pb toxicity significantly inhibited all the photosynthetic attributes, that is, net photosynthetic rate, stomatal conductance, internal CO_2_ concentration, and transpiration rate as compared to control, and this inhibition was recognized as dose-dependent. Exogenous application of ALA alone exhibited a significant positive effect on stomatal conductance and transpiration rate of the rapeseed plants. The ALA at 25 mg L^−1^ increased the net photosynthetic rate by 10.03% and 44.72%, stomatal conductance by 33.33% and 41.93%, internal CO_2_ concentration by 15.32% and 27.36%, and transpiration rate by 53.57% and 74.02% under different levels of Pb (100 and 400 *μ*M, resp.) as compared to their respective controls.

### 3.3. ALA Reduced Pb Uptake in Brassica Plants under Pb Stress

Pb concentration was significantly enhanced in the shoots and roots of *B. napus* plants under Pb alone conditions (Table 4). The magnitude of Pb uptake was increased, as the Pb level increased in solution culture, and uptake of Pb contents was more in roots as compared to shoots under different Pb concentrations. Foliar spray of ALA significantly reduced the Pb contents in both shoots and roots under different Pb concentrations. The ALA at 25 mg L^−1^ reduced the Pb contents in shoots by 38.88% and 61.25% and in roots by 70.07% and 72.80% under different concentrations of Pb (100 and 400 *μ*M, resp.) as compared to their respective controls.

### 3.4. ALA Alleviated the Pb-Induced Ultrastructural Changes

**Figure 2:**
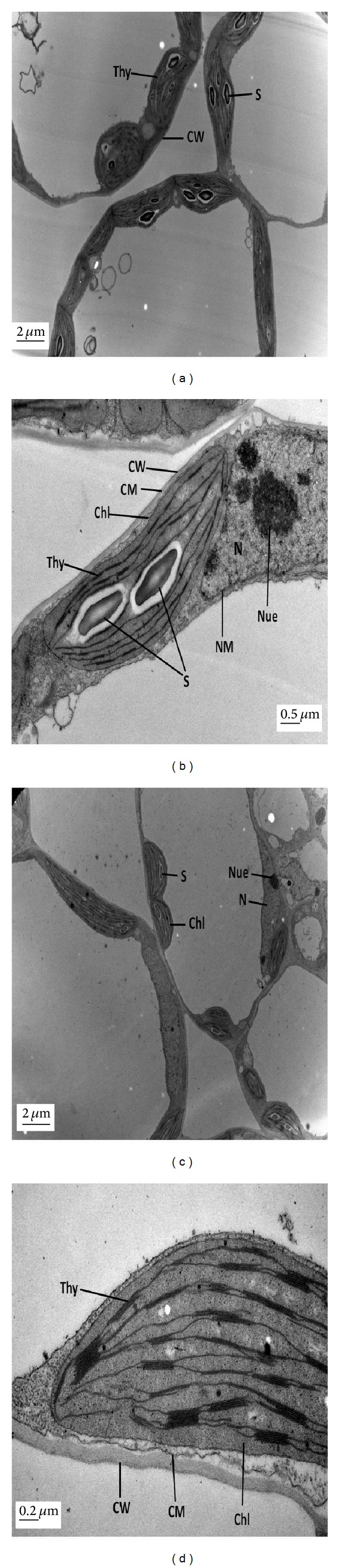
Electron micrographs of leaf mesophyll cells of 15-day hydroponically grown seedlings of *Brassica napus* cv. ZS 758. (a-b) TEM micrographs of mesophyll cells of leaves of ZS 758 at control level at low and high magnifications, respectively, show a clear cell wall (CW) and cell membrane (CM), well-developed chlorophyll (Chl) with thylakoids, grana, and starch grain (S). (c-d) TEM micrographs of mesophyll cells of ZS 758 at 25 mg L^−1^ 5-aminolevulinic acid (ALA) alone at low and high magnifications, respectively, show a small nucleus (N) with developed nucleolus (Nue) and nuclear membrane (NM), well-developed chloroplast (Chl) with thylakoids, grana, and starch grains (S). Cell wall (CW) and cell membrane (CM) are present. Moreover, intercellular spaces are found.

**Figure 3:**
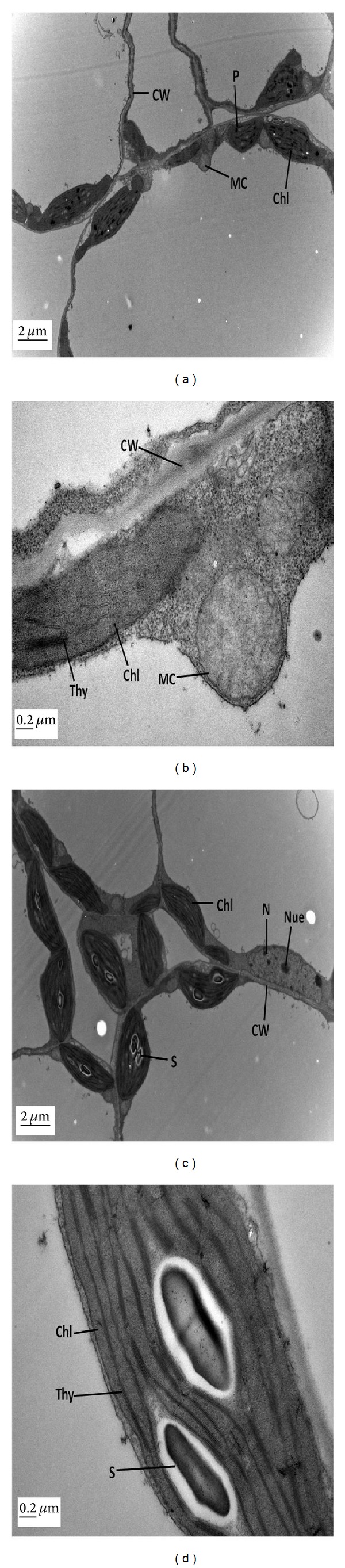
Electron micrographs of leaf mesophyll cells of 15-day hydroponically grown seedlings of *Brassica napus* cv. ZS 758. (a-b) TEM micrographs of mesophyll cells of ZS 758 under 400 *μ*M lead (Pb) at low and high magnifications, respectively, show swollen and ruptured chloroplast (Chl) and absence of starch grain (S). Cell wall (CW) and cell membrane (CM) are diffused with each other and no nucleus was found. Moreover, accumulation of Pb is increased in the form of electron dense granules attached to the cell walls. (c-d) TEM micrographs of mesophyll cells of ZS 758 at 25 mg L^−1^ 5-aminolevulinic acid (ALA) under 400 *μ*M lead (Pb) at low and high magnifications, respectively, show an elongated nucleus (N) with well developed nucleolus (Nue) and nuclear membrane (NM). Cell wall (CW) and cell membrane (CM) are clear. Chloroplast (Chl) is well-developed in almost round shape and has thylakoids, grana, and big starch grain (S).

**Figure 4:**
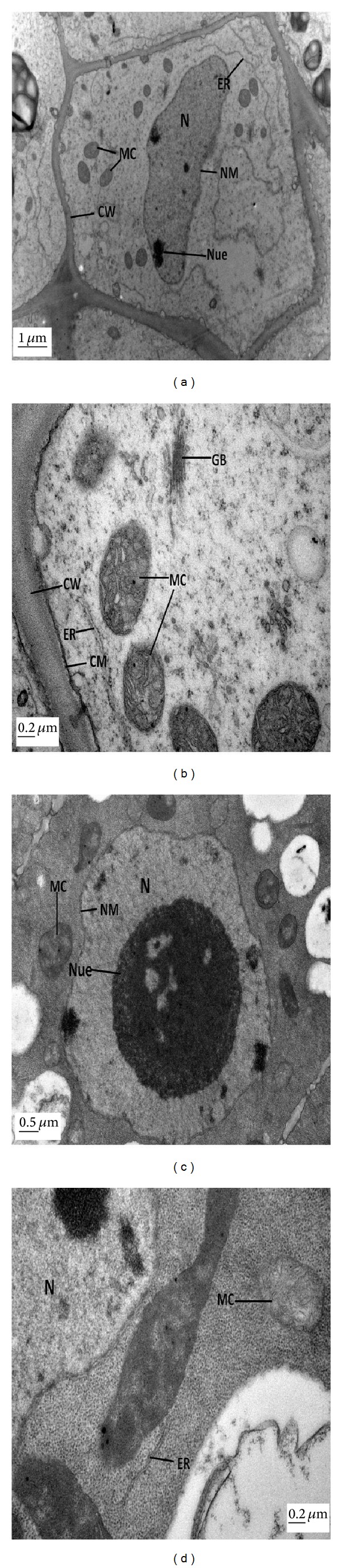
Electron micrographs of root tip cells of 15-day hydroponically grown seedlings of *Brassica napus* cv. ZS 758. (a-b) TEM micrographs of root tip cells of ZS 758 at control level at low and high magnifications, respectively, show well-developed nucleus (N) with nucleolus, smooth cell wall (CW), clear endoplasmic reticulum (ER), golgi bodies (GB), and well-developed mitochondria (MC). (c-d) TEM micrographs of root tip cells of ZS 758 at 25 mg L^−1^ 5-aminolevulinic acid (ALA) alone at low and high magnifications, respectively, show a clear cell with well-developed nucleus (N) with nucleoli (Nue) and a distinct nuclear membrane, a number of well-developed mitochondria (MC), and continuous endoplasmic reticulum (ER).

**Figure 5:**
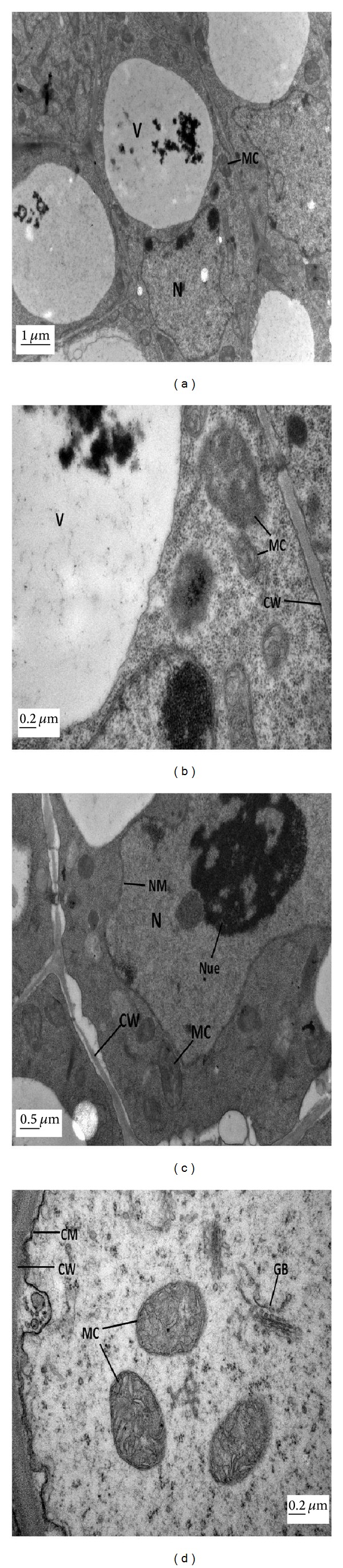
Electron micrographs of root tip cells of 15 day-hydroponically grown seedlings of *Brassica napus* cv. ZS 758. (a-b) TEM micrographs of root tip cells of ZS 758 at 400 *μ*M Pb alone at low and high magnifications, respectively, show undeveloped nucleus (N) with broken nuclear membrane (NM), undeveloped mitochondria (MC), disappearance of endoplasmic reticulum (ER), and cracked cell wall (CW). Moreover, Pb accumulation increased in the form of electron dense granules along the cell wall and vacuole (V). (c-d) TEM micrographs of root tip cells of ZS 758 at 25 mg L^−1^ 5-aminolevulinic acid (ALA) under 400 *μ*M Pb concentration at low and high magnifications, respectively, show a clear cell with big nucleus (N), number of developed mitochondria (MC), golgi bodies (GB), and cell wall (CW).

Ultrastructural changes in leaf mesophyll and root tip cells of *B. napus* L. (cv. ZS 758) are demonstrated in Figures 2–5. The TEM micrographs of leaf mesophyll cells of ZS 758 at the control level (i.e., untreated) at low and high magnification are shown in Figures 2(a) and 2(b). There was clear chloroplast with smoothly packed granum and thylakoid membranes along with a number of starch granules. The cell wall and cell membrane were also clear and apparent. The cell also contained a clear nucleus with nucleolus and nuclear membrane. A whole mesophyll cell was observed at the level of 25 mg L^−1^ ALA alone as depicted in Figures 2(c) and 2(d). There were maximum intercellular spaces and all the organelles in the cells were clearly differentiated. A clear and well-developed cell wall and cell membrane could be observed in the micrographs. The nucleus was present with a nucleolus and a clear nuclear membrane. There was a well-developed chloroplast with packed granum and thylakoid membranes. The TEM micrographs of leaf mesophyll cells of ZS 758 at Pb (400 *μ*M) alone at low and high magnification are shown in Figures 3(a) and 3(b). The higher concentration of Pb (400 *μ*M) totally damaged the mesophyll cell and reduced the intercellular spaces between all the cells. The cell wall and cell membrane were fused to each other. The higher concentration of Pb totally damaged the thylakoid membranes of chloroplast. There were no more starch granules in the chloroplast as compared to controls. The changes in the fine structures of leaf mesophyll cells due to foliar application of 25 mg L^−1^ ALA under 400 *μ*M Pb have been highlighted in Figures 3(c) and 3(d). The nucleus was well developed with a nuclear membrane and nucleolus. The intercellular spaces among the cells could be seen in the micrograph. The chloroplast was clear having a number of starch granules. Briefly, it was observed that a high dose of Pb ruptured the structures of all organelles and the starch granule was not present. The application of ALA decreased the drastic effect of Pb stress in the leaf ultrastructures of rapeseed plants in the present study.

The TEM of root cells of ZS 758 at control treatment with low and high magnifications have been shown in Figures 4(a) and 4(b). The micrograph showed a clear nucleus with nuclear membrane and nucleolus. Moreover, clear endoplasmic reticulum and mitochondria were found in root tip cell. The cell wall and cell membrane were smooth and continuous, and golgi bodies can also be observed in the micrograph. A big and round shape nucleus was observed with nucleolus and nuclear membrane at the level of 25 mg L^−1^ ALA alone (Figures 4(c) and 4(d)). A well-developed mitochondria and endoplasmic reticulum were found in micrographs. The TEM of root cells of ZS 758 at Pb (400 *μ*M) alone at low and high magnification are shown in Figures 5(a) and 5(b). Pb concentration at 400 *μ*M showed obvious ultrastructural changes as compared to control. There was an elongated nucleus with immature nucleolus. The cell wall and cell membrane were diffused into each other and mitochondrion was undeveloped. Pb was present in the form of small granules in the vacuole and along the cell wall. The changes in the fine structures of root tip cells due to foliar application of 25 mg L^−1^ ALA under 400 *μ*M Pb have been depicted in Figures 5(c) and 5(d). There was well-developed nucleus with nuclear membrane in the cell. The ALA alleviated the Pb stress and presented clear mitochondria, cell wall, and golgi bodies in root tip cell.

## 4. Discussion

Lead (Pb) is a highly phytotoxic heavy metal in the environment causing alteration in various physiological processes and ultrastructural changes in plants [7]. Generally, the sensitivity to heavy metal of a given plant depends on metal concentration, dose duration, plant variety and type, age of plant, and plant tissue being analyzed [25]. The present study was carried to analyze the beneficial role of 5-aminolevulinic acid (ALA) on oilseed *B. napus* plants under Pb stress. The present results demonstrated that Pb toxicity visibly inhibited the plant growth parameters (Table 1). The reduction in plant growth would be the adverse effect of Pb on physiological processes and disturbance in plant mineral nutritional status. Moreover, reduction in root growth might be due to Pb-induced inhibition of cell division in root cell [7]. On the other hand, plant growth was improved under the combined treatment of ALA and Pb stress (Table 1). This increase in growth with ALA might be due to the fact that ALA has a promotive role in regulating different metabolic processes, thereby improving growth and yield of most plants under abiotic stresses [26]. Recently, it has been recognized that ALA enhanced the plant growth by alleviating cadmium effects in *B. napus* [12].

The data showed that Pb toxicity significantly reduced the root morphology of plants (Table 2). The possible reason behind is that Pb toxicity leads to the imbalance of water status and disturbed nutrient uptake and inhibits the cell division in the root tip cell [7]. Previously, it was observed that Pb toxicity demoted the root morphology in two ecotypes of *Elsholtzia argyi *[3]. Schwarz and Grosch [27] also conducted similar studies to discover the effects of Pb on the relationship between yield and root morphology. Meanwhile, when plant sustained foliar application of ALA, it significantly improved the root morphology of *B. napus* plants under the Pb stress (Table 2). This improvement in root morphology under the application of ALA might be due to the fact that ALA activates the heme-based antioxidant systems (APX, POD, and CAT) to scavenge the reactive oxygen species like H_2_O_2_ [18, 28]. Moreover, this study showed that Pb stress alone reduced the chlorophyll contents (Table 3) and photosynthetic parameter as compared to control (Figure 1). This reduction in photosynthetic attributes might be due to the destruction of protein complex, chloroplast, and photosynthetic apparatus under metal stress [29], and at the same time heavy metal stress might inhibit the photosynthetic electron transport chain [30]. Moreover, decomposition of chlorophyll might be due to increase in chlorophyllase activity under heavy metal stress [31]. It has been well documented in the literature that heavy metals can affect stomatal conductance, gas exchange, and chlorophyll contents, thereby affecting the net photosynthetic rate [32]. At the same time, increase in chlorophyll and net photosynthesis rate with foliar application of ALA confirms that ALA performs as growth regulator and enhances the parameters involved in photosynthesis. The role of ALA in improving photosynthetic attributes can therefore be due to the significant improvement in Chl content [33] and subsequently increasing light harvesting capacities in the treated plants. Ali et al. [12] found that foliar application of ALA significantly elevated the photosynthetic parameters in *B. napus* plants under cadmium stress conditions.

The results showed that Pb contents were increased in different plant parts with increasing Pb concentration in the medium (Table 4). These results about more uptake of Pb contents in roots than shoots express the capability of plants to avoid metal-induced changes [34]. In this study, it was found that foliar application of ALA reduced the uptake of Pb in different parts of plants under Pb stress. It may be due to the application of ALA which strengthens the plant defense system by activating antioxidant machinery, thus prevents the uptake or movement of heavy metal in plant tissues [12, 13]. So, it can be suggested that exogenous ALA could be applied for the purpose of phytoextraction.

Microscopic examination was performed as it helped to study the changes at cellular level [35]. In the present study, plant mesophyll cell and root tip cells were significantly damaged under the Pb toxicity alone conditions. It was advocated that chloroplasts are highly susceptible to oxidative stress [36]. In mesophyll cell, Pb-induced stress brought about the disappearance of starch granules inside the chloroplast and dissolved thylakoids membranes (Figures 3(a) and 3(b)). Under Pb stress, metal deposition was present in vacuoles and cell walls and all the organelles were totally damaged in root tip cells (Figures 5(a) and 5(b)). Previously, similar ultrastructural disorders in plant cells were noticed under heavy metal stress [37]. However, foliar application of ALA improved these structures under Pb toxicity in both mesophyll cell (Figures 3(c) and 3(d)) and root tip cell (Figures 5(c) and 5(d)). Improved cell structures with foliar application of ALA under Pb-treated plants could be the indication of less oxidative stress and might be due to that ALA helped in inducing the antioxidant system in the thylakoids and cell membranes. The higher activity of ascorbic peroxidases catalyzed the breakdown of H_2_O_2_ in Halliwell-Asada pathway originally described in the chloroplast [38]. So, it can be suggested that activity of ascorbic peroxidases can potentially protect thylakoids from activated oxygen species under the Cd stress. Naeem et al. [39] and Ali et al. [13, 18] also observed that ALA improved the cell structures in *B. napus* under the salinity and cadmium stress, respectively. Previously, Castelfranco and Jones [40] suggested that ALA showed alleviating role to trigger the heme-based molecules. It can be concluded that ALA may alleviate oxidative stress induced by Pb toxicity. From the present findings, it could be suggested that ALA ameliorated the Pb-induced inhibition of plant growth, photosynthesis, and ultrastructural changes in plant cells.

## 5. Conclusions

According to the present study, we observed that Pb reduced the plant growth and root morphology as well as damaged the cell ultrastructures. Meanwhile, exogenously applied ALA increased the plant growth and improved cell ultrastructures under the Pb toxicity. Overall, results from present research reveal that ALA has the ability to get rid of the adverse effects of Pb on *B. napus* by increasing plant growth and net photosynthesis rate and improving the ultrastructural changes induced by Pb stress. To examine the precise ameliorative role of ALA under Pb stress conditions a soil environment-based approach is needed.

## Figures and Tables

**Table 1:** Effects of treatments of 5-aminolevulinic acid (ALA) (mg L^−1^) and lead (Pb) (*μ*M) on plant height (cm), stem length (cm), root length (cm), and leaf area (cm^2^ plant^−1^) of *Brassica napus* L.

ALA conc.	Pb conc.	Mean values and their relative increase/decrease over respective controls			
		Plant height	Stem length	Root length	Leaf area
0	0	44.73 ± 2.83^a^ (0)	14.46 ± 0.94^a^ (0)	18.22 ± 1.17^b^ (0)	325.66 ± 14.62^a^ (0)
	100	37.47 ± 2.53^b^ (−16.22)	9.58 ± 0.71^c^ (−33.74)	17.54 ± 1.27^bc^ (−3.73)	223.09 ± 14.59^c^ (−31.49)
	400	26.73 ± 2.11^c^ (−40.24)	6.35 ± 0.58^d^ (−56.08)	11.55 ± 0.94^d^ (−36.61)	174.25 ± 11.53^d^ (−46.49)

25	0	45.35 ± 2.69^a^ (+1.38)	13.50 ± 0.81^ab^ (−6.06)	18.65 ± 1.31^b^ (+2.36)	333.14 ± 12.98^a^ (+2.31)
	100	42.50 ± 2.06^a^ (+13.42)	12.26 ± 0.79^b^ (+27.97)	21.49 ± 1.73^a^ (+22.51)	312.90 ± 13.65^a^ (+40.25)
	400	37.51 ± 1.00^b^ (+40.32)	10.42 ± 0.73^c^ (+64.09)	15.39 ± 1.03^c^ (+33.24)	265.66 ± 13.27^b^ (+52.45)

Values are the means of four replications ± SD. Variants are significant at *P* ≤ 0.05.

**Table 2:** Effects of treatments of 5-aminolevulinic acid (ALA) (mg L^−1^) and lead (Pb) (*μ*M) on the root morphology of *Brassica napus* L.

ALA conc.	Pb conc.	Mean values and their relative increase/decrease over respective controls			
		Root diameter (cm)	Root surface area (cm^2^)	Root volume (cm^3^)	Number of root tips
0	0	4.92 ± 0.17^bc^ (0)	237.44 ± 13.15^b^ (0)	12.76 ± 0.80^b^ (0)	1329.83 ± 21.59^c^ (0)
	100	4.09 ± 0.12^d^ (−16.86)	213.85 ± 14.11^c^ (−9.93)	11.45 ± 0.58^c^ (−10.26)	1293.25 ± 21.78^c^ (−2.75)
	400	2.58 ± 0.11^e^ (−47.56)	167.48 ± 8.61^d^ (−92.63)	7.62 ± 0.66^d^ (−40.28)	898.61 ± 19.07^e^ (−32.42)

25	0	5.34 ± 0.18^a^ (+8.53)	264.71 ± 11.83^a^ (+11.48)	13.11 ± 0.37^b^ (+2.74)	1392.99 ± 20.05^b^ (+4.74)
	100	5.11 ± 0.10^ab^ (+24.93)	249.68 ± 12.53^ab^ (+16.75)	14.99 ± 0.69^a^ (+30.91)	1488.33 ± 19.35^a^ (+15.08)
	400	4.74 ± 0.23^c^ (+83.72)	193.78 ± 11.33^c^ (+15.70)	10.68 ± 0.61^c^ (+40.15)	1150.03 ± 24.55^d^ (+27.97)

Values are the means of four replications ± SD. Variants are significant at *P* ≤ 0.05.

**Table 3:** Effects of treatments of 5-aminolevulinic acid (ALA) (mg L^−1^) and lead (Pb) (*μ*M) on chlorophyll and carotenoids contents (mg g^−1^ FW) in the leaves of *Brassica napus* cv. ZS 758.

ALA conc.	Pb conc.	Mean values and their relative increase/decrease over respective controls			
		Chlorophyll a	Chlorophyll b	Total chlorophyll	Carotenoids
0	0	47.92 ± 2.56^a^ (0)	58.54 ± 1.64^b^ (0)	106.47 ± 1.87^b^ (0)	4.89 ± 0.81^ab^ (0)
	100	44.45 ± 1.98^a^ (−7.24)	58.31 ± 1.51^b^ (−0.39)	102.76 ± 3.50^b^ (−3.74)	4.87 ± 0.59^abc^ (0)
	400	34.20 ± 1.94^b^ (−28.63)	47.50 ± 1.88^d^ (−18.85)	81.71 ± 1.11^d^ (−23.25)	3.54 ± 0.37^c^ (−27.31)

25	0	47.48 ± 2.25^a^ (−0.91)	64.93 ± 1.76^a^ (+10.91)	112.42 ± 4.01^a^ (+5.58)	5.43 ± 0.60^a^ (+11.49)
	100	45.23 ± 2.35^a^ (+1.75)	61.40 ± 1.71^b^ (+5.31)	106.64 ± 4.06^b^ (+3.77)	4.92 ± 0.48^ab^ (+1.02)
	400	37.63 ± 2.27^b^ (+10.02)	53.73 ± 1.75^c^ (+13.11)	91.42 ± 2.89^c^ (+11.88)	3.95 ± 0.42^bc^ (+11.58)

Values are the means of four replications ± SD. Variants are significant at *P* ≤ 0.05.

**Table 4:** Effects of treatments of 5-aminolevulinic acid (ALA) (mg L^−1^) and lead (Pb) (*μ*M) on Pb contents in shoots and roots of *Brassica napus *cv. ZS 758.

ALA conc.	Pb conc.	Shoot (mg kg^−1^ DW)	Root (mg kg^−1^ DW)
0	0	0.34 ± 0.01^e^	0.65 ± 0.03^d^
	100	144.84 ± 9.50^c^	756.44 ± 8.67^b^
	400	461.79 ± 13.02^a^	2741.12 ± 16.90^a^

25	0	0.27 ± 0.01^e^	0.73 ± 0.02^d^
	100	88.58 ± 7.14^d^	226.35 ± 13.14^c^
	400	178.93 ± 15.35^b^	745.54 ± 9.50^b^

Values are the means of four replications ± SD. Variants are significant at *P* ≤ 0.05.
